# Lactoferrin: A Promising Therapeutic Molecule against Human Papillomavirus

**DOI:** 10.3390/nu16183073

**Published:** 2024-09-12

**Authors:** Merve Kaplan, Merve Baktıroğlu, Arda Erkan Kalkan, Ahmet Alperen Canbolat, Mauro Lombardo, António Raposo, José Luiz de Brito Alves, Anna Maria Witkowska, Sercan Karav

**Affiliations:** 1 Theoretical and Physical Chemistry Laboratory, Department of Chemistry, University of Oxford, Oxford OX1 2JD, UK; merve.kaplan@chem.ox.ac.uk; 2 Department of Molecular Biology and Genetics, Canakkale Onsekiz Mart University, Canakkale 17100, Turkey; ardaerkankalkan@gmail.com (A.E.K.); ahmetalperencanbolat@stu.comu.edu.tr (A.A.C.); 3 Department of Gynecological Oncology, Istanbul University, Istanbul 34452, Turkey; mervebak_1987@hotmail.com; 4 Canakkale Mehmet Akif Ersoy Government Hospital, Canakkale 17110, Turkey; 5 Department for the Promotion of Human Science and Quality of Life, San Raffaele Open University, Via di 11 Val Cannuta 247, 00166 Rome, Italy; mauro.lombardo@uniroma5.it; 6 CBIOS (Research Center for Biosciences and Health Technologies), Universidade Lusófona de Humanidades e Tecnologias, Campo Grande 376, 1749-024 Lisboa, Portugal; antonio.raposo@ulusofona.pt; 7 Department of Nutrition, Health Science Center, Federal University of Paraíba, João Pessoa 58051-900, PB, Brazil; jose.luiz@academico.ufpb.br; 8 Department of Food Biotechnology, Bialystok Medical University, 15-089 Bialystok, Poland

**Keywords:** lactoferrin, natural compound, dairy foods, milk, antiviral, human papillomavirus, HPV

## Abstract

Lactoferrin is a multifunctional glycoprotein naturally found in mammalian secretions, predominantly in colostrum and milk. As a key component of dairy foods, lactoferrin enhances viral protection and boosts human health, owing to its fundamental properties including antiviral, anti-inflammatory, and immune-modulatory effects. Importantly, the antiviral effect of lactoferrin has been shown against a range of viruses causing serious infections and threatening human health. One of the viruses that lactoferrin exerts significant antiviral effects on is the human papillomavirus (HPV), which is the most prevalent transmitted infection affecting a myriad of people around the world. Lactoferrin has a high potential to inhibit HPV via different mechanisms, including direct binding to viral envelope proteins or their cell receptors, thereby hindering viral entry and immune stimulation by triggering the release of some immune-related molecules through the body, such as lymphocytes. Along with HPV, lactoferrin also can inhibit a range of viruses including coronaviruses and hepatitis viruses in the same manner. Here, we overview the current knowledge of lactoferrin and its effects on HPV and other viral infections.

## 1. Introduction

Dairy milk and its nutritional content, in terms of valuable bioactive proteins, exert a diverse function ranging from antiviral to immunomodulatory [1,2,3,4,5,6]. Lactoferrin or in short, Lf, is an important whey protein in most mammals’ milk, various dairy milk, and other dairy-related products [3,4,7]. Lf offers diverse functions ranging from antiviral to anticancer effects due to its unique structure and characteristics [4]. It is an 80 kDa protein including two different subunits named *N*- and *C*-lobes, which can bind to distinct metals such as Fe^2^⁺ and Zn^2^⁺ [5]. Importantly, Lf can retain iron at an acidic pH where infection and inflammation take place with its cationic nature, enabling it to bind negatively charged viral particles and viral receptors on the cell membrane, such as heparan sulfate (HS) chains attached to the cell membrane via protein chains [6,8]. Thus, it is commonly considered a therapeutic glycoprotein against viral infections as it directly provides inhibition or alleviates the inflammatory-related mechanisms and supports host immunity [9,10,11,12,13]. Following the significant effects of Lf on distinct viral infections to date, it has a high potential to be an effective agent against a widespread viral infection: human papillomavirus (HPV). HPV is currently the most prevalent sexually transmitted infection (STI). It has hundreds of subtypes and severely affects millions of people worldwide [14,15]. Importantly, some distinct strains of HPV, such as HPV-16 and HPV-18, might be carcinogenic [16] and develop into cervical cancer which causes thousands of deaths annually [17]. The antiviral potential of Lf is commonly attributed to three main mechanisms: First, Lf can directly bind to the viral particles, predominantly, the viral envelope proteins, by using its complex and heterogeneous glycans, and so it prevents viral binding to host receptors. Secondly, Lf can directly attach to HPV receptors, such as HS chains on the cell membrane, which inhibits viral entry mechanisms [8]. The third mechanism is related to an indirect way in which Lf stimulates immunity molecules such as chemokines and lymphocytes that can inhibit viral replication in the host by enhancing host immunity [18]. Different in vitro and in vivo studies in this research show that Lf acts as a therapeutic protein for HPV infection and other crucial viral infections, from COVID-19 to hepatitis B [19,20,21]. All this information underscores why Lf is widely recognized for its relevance in combating HPV. Lf effectively binds to viral particles and receptors on host cells, inhibiting viral entry and replication. It is also acknowledged for stimulating the immune system and enhancing immune responses to manage HPV infections. Its effectiveness in treating HPV and reducing the risk of HPV-related diseases, such as cervical cancer, is well-established. Therefore, Lf is considered a valuable therapeutic and preventive agent for HPV-related conditions in the global clinical market. Here, we explore the potential effect of Lf as a dairy food component on major viral infections, primarily HPV, highlighting the mechanisms of action and implications for future research and medical applications targeting this crucial glycoprotein.

## 2. Lactoferrin: Structure and Biological Functions

Lf is a highly glycosylated and multifunctional protein of the transferrin family [22]. It was first identified and purified from cow milk in 1939 and then discovered also in tears, saliva, mucus, skin, semen, and white blood cells [3,23,24]. Lf is synthesized by many mammals including bovine, human, horse, goat, and some rodent types [24,25,26]. Lf includes about 700 amino acids with a molecular weight of about 80 kDa and has an isoelectric point of 8–8.5 [27,28]. The glycosylation of Lf is highly variable in terms of the abundance and location of the glycosylation sites depending on the species. For instance, human Lf has three glycosylation sites, Asn-137, 478, and 623, whereas bovine Lf possesses five glycosylation sites including Asn-233, 281, 368, 476, and 545. The glycosylation level and location are critical for Lf sensitivity to proteolysis and its physiological functions [29]. Additionally, Lf has two lobes, N (1–332 amino acid) and C (344–703 amino acid), which show a high homology of about 33–43 percent [5]. Each of these lobes can bind diverse metal ions, especially Fe^2+^ and Fe^3+^ with a carbonate ion (CO_3_^2−^). The three-dimensional structure of Lf demonstrates that CO_3_^2−^ binds first and, therefore, the positive charge of Arg is neutralized. The CO_3_^2−^ in the iron coordination binding is critical for iron reversible binding since CO_3_^2−^ protonation is the first step in the breaking of the iron site under a low pH [30,31]. Regarding the binding of Fe, Lf has two conformations called apo and holo forms. The difference between these two forms is that apo-Lf does not include any bound Fe^3+^ ion, but holo-Lf is in the form of a complex bound with Fe^3+^. As holo-Lf includes conjugated Fe^3+^, it has a closed structure that enables it to resist proteolysis more efficiently than apo-Lf with an open conformation [32]. The iron-binding ability of Lf significantly contributes to its biological properties since the iron ion is an important player in many physiological functions, such as cell homeostasis and microorganism growth. Because of the unique structural characteristics of Lf, it is involved in different biological functions, from immune response to viral disease defense [4]. This versatile glycoprotein has attracted regard to the serious pandemic in 2019 called COVID-19 due to its high antiviral effects [33]. Considerably, the antiviral, anti-inflammatory, and immune-modulatory functions enable Lf to be an effective therapeutic protein against different diseases, from viral infections to cancer, by boosting immunity and preventing host cells.

One of the significant effects of Lf bioactivity is related to its immune-modulatory and anti-inflammatory functions [34,35,36]. It serves various functions, namely dendritic cell maturation, triggering proliferation, and declining antigen internalization to enhance immunity [37,38]. In the context of dendritic cell (DC) differentiation, Lf inhibits their mechanism by hindering their responsiveness towards TLR ligands. It is also shown that these effects are diminished when the protein is degraded, indicating that the LF-induced differentiation of monocytes into hyporesponsive DCs is not mediated by endotoxin tolerance. LF may take a role in promoting immune homeostasis in the GI tract [39]. In some reported in vitro model studies with different types of intracellular microorganisms [40,41,42], Lf in both the apo- or iron-saturated form exerts an inhibitory effect against the microbial internalization. The ability of Lf to bind to the GAGs of host cells seems to have crucial implications in the inhibition of bacterial internalization [43]. One of the reported studies indicates that the binding of Lf to integrins through the domains targeted by invasin and to GAGs may induce a dramatic collapse in bacterial–host cell interactions. This mechanism inhibits bacterial internalization [40]. The main mechanism of Lf immunomodulation is related to stimulating the production of immunity-related cells such as T-lymphocytes and B-lymphocytes. In a related study, it was observed that mice, in which bovine Lf was orally administered, had developed a strongly elevated pool of CD3 + T and CD4 + T cell content as a response [44]. Furthermore, CP-immunosuppressed mice given Lf orally could reconstitute a T cell-mediated immune response by regenerating the T cell pool [45]. Lf can also induce the differentiation of T cells from their immature precursors via the induction of the expression of the CD4 antigen under non-pathogenic conditions [46,47]. On the other hand, in the context of a humoral response, Lf was demonstrated to promote the differentiation of splenic B cells in vitro [48]. In addition, Lf binding to CpG-containing oligonucleotides was shown to inhibit their immunostimulatory effects on human B cells [49]. Additionally, Lf can help to activate some lymphocytes including B, T, and NK in the spleen, peripheral blood, and intestine [50,51]. Consequently, Lf can modulate the activity of T and NK cells via the proliferation of T cells [52]. Another noticeable point is that Lf can activate macrophages by stimulating TLR4-dependent and independent signaling that also enhances the production of interleukin 6 (IL-6) and CD-40 [53].

Such inflammation- and immunity-related effects of Lf are particularly highlighted in the context of viral infections, with a specific emphasis on diseases such as COVID-19. Notably, in instances of viral infections, the severity of the disease is often influenced by the degree of the immune response and subsequent inflammation [34]. This holds particular significance in the case of COVID-19, as it has been identified that hyperinflammation in humans is primarily initiated by cytokine storm syndrome rather than the viral infection itself [34]. In severe cases of COVID-19, a distinctive cytokine profile emerges, characterized by elevated levels of cytokines and acute-phase reactants such as IL-6, tumor necrosis factor-alpha (TNFα), and ferritin. In this context, Lf has been shown to reduce IL-6 and TNFα levels and downregulate ferritin expression in sepsis-simulating experimental settings [54,55]. Therefore, the anti-inflammatory effect of bovine Lf results in a decrease in viral replication, as evidenced by in vitro models infected with SARS-CoV-2 [56]. Additionally, Lf strongly interacts with the gp120 protein of HIV by binding to its DC-SIGN receptor on dendritic cells and hinders their interactions, which is essential for the HIV entry mechanism. Thereby, the transmission of the virus through the host cell is inhibited by Lf [26,57]. Overall, Lf is a vital anti-inflammatory and immune-modulatory agent and employs its effect by using different pathways to prevent the host from viral entry and, therefore, infection.

## 3. Antiviral Effect of Lactoferrin

**Figure 1:**
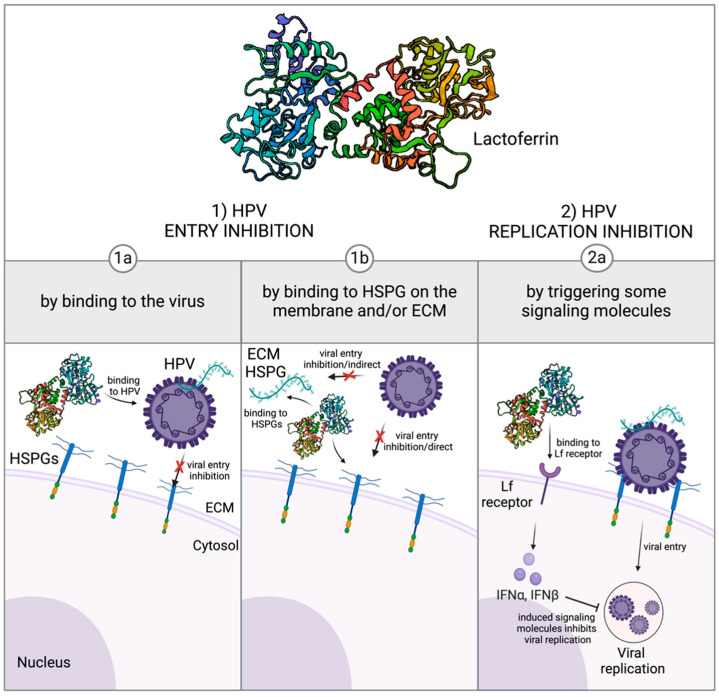
Potential antiviral effects of lactoferrin (PDB 1BLF) on papillomavirus. Lactoferrin can inhibit viral infection in its early stages by suppressing viral entry and/or inhibiting viral replication in further stages. It can directly attach to viral glycans found on their membranes and inhibit their binding to cell surface receptors (1a). It can also inhibit viral entry by binding to HSPGs, which are found on the cell membrane or free in the ECM, that are responsible for the viral entry mechanism (1b). It can bind to its receptors on the cell membrane and trigger some interferons that can prevent viral replication (2a) [6,8,18] (Created with BioRender.com).

The antiviral effect of Lf is mainly attributed to the interactions between the cationic nature of Lf and host cell glycosaminoglycans (GAGs), which are negatively charged and complex oligosaccharides playing a role in many biological functions in the human body (Figure 1). As one of the functions of GAGs is guiding viral entry through host cells, the virus invasion is inhibited when Lf binds to these sugar chains. Lf can inhibit viral progress, especially by binding to HS conjugated to the protein core on the host cell membrane to form heparan sulfate proteoglycans (HPSGs). HS is commonly considered the coreceptor or cofactor for different types of viruses including SARS-CoV-2. Since Lf can bind to these receptors like viruses, the entry mechanism of viruses through the host cell is inhibited [6,8,58]. Additionally, Lf can trigger α and β interferon (IFN) expression by intracellular signals and Lf receptors, which also hinders viral replication inside the host cell (Figure 1) [18].

By these mechanisms of action, the antiviral effect of Lf has been proven against a variety of virus types such as HPV, herpes simplex virus I-II (HSV-1 and HSV-2), hepatitis C and B virus, human immunodeficiency virus (HIV), and SARS-CoV-2 [59,60,61,62,63,64,65,66]. For instance, diverse mechanisms of action have been suggested for Lf antiviral activity against coronaviruses including SARS-CoV-2, such as interactions with the spike protein, binding to HSPGs on the cell membrane, and enhancing interferon response [8,56]. It has been reported that the Lf level was significantly lower in the saliva samples of patients with SARS-CoV-2 in comparison to the healthy controls [67]. It was also shown that bovine Lf is effective in preventing SARS-CoV-2 and some other coronaviruses such as HCoV-OC43, HCoV-229E, and HCoV-NL63 entry in a variety of cell lines including Vero E6, 293T-ACE2, and Calu-3 by binding to HPSGs. Even more, bovine Lf dose-dependently blocked SARS-CoV-2 viral replication and the generation of mature virions at the attachment level in viral progress [8]. Recently, it has been demonstrated that liposomal encapsulation of bovine Lf showed a more potent antiviral effect against SARS-CoV-2 and HCoV-229E in comparison to the free form in Huh-7 cells [33]. Lf showed its high antiviral activity not only in coronaviruses but also in other different viruses. For instance, Marchetti and his colleagues revealed that bovine Lf inhibited HSV-1 infection in cells expressing HS and/or another GAG chondroitin sulfate which aids HSV-1 entry through the host, whereas a low inhibitory effect was observed in GAG deficient cells. This indicates that the antiviral effect of Lf against HSV-1 is dependent on its interaction with GAGs on the cell membrane [61]. Moreover, Lf exerts a synergy with some other antiviral molecules, including ribavirin and interferon for hepatitis C treatment. A higher oral intake of bovine Lf at 3.6 g for 24 weeks with ribavirin and interferon caused a significant increment in the antiviral effect [68]. Accordingly, Lf, as an effective antiviral agent of dairy milk, has caused the inhibition of distinct viral infections from HSV to COVID-19.

### 3.1. Lactoferrin against HPV

Lf, as a multifunctional protein and key component of dairy foods, has a high potency to inhibit HPV, which might prevent its transformation to cervical cancer. Several studies have suggested that Lf inhibits HPV binding and entry through host cells [59,69,70]. HPV, the most prevalent transmitted infection, affects over 300 million people in the world with 400 distinct subtypes [14,15]. Most types of HPV strains affect mucosal tissues and the genital tract [71]. Regarding HPV symptoms, it commonly causes larynx papilloma, condyloma acuminata, and skin warts. Genital-based HPV types differ in terms of their risk level for cancer development [72]. In further stages, some strains of HPV might be highly carcinogenic (e.g., HPV16 and HPV18) and cause lesions in the host [16,17]. Importantly, HPV-related cervical cancer is a serious health issue worldwide, causing more than 300,000 deaths every year [17]. Hence, understanding the HPV genome, the mechanism of its infection, and how it transforms its way to cervical cancer is critical for the development of new potential therapeutic applications. HPV is a circular, double-stranded DNA virus and consists of nine open reading frames that encode seven early genes from E1 to E7. Additionally, every HPV capsid includes two late genes (L1 and L2) with a standard ratio of 5:1 (L1:L2) [73,74]. Early genes have mainly regulatory functions in infected cells, whereas late genes encode capsid proteins. The HPV genome includes long control regions with regulatory frames that are involved in post-transcription and replication [75,76]. Regarding cervical tissue, each HPV region is found in different locations. The early genes, for example, are in the basal layers and the first viral replication occurs due to E1–E2 integration. Specifically, E6 can inactivate tumor suppressor protein p53 involved in DNA repair and E7 also silences some genes in tumor suppression (e.g., pRb and p130). This indicates that E6 and E7 proteins take part in important roles in the transformation of the viral infection into cervical cancer [77]. On the other hand, late genes commonly play a role in capsid conformation changes, gene regulation, and morphogenesis [78]. For instance, the L2 protein is associated with the modulation of mRNA splicing in the epithelium, which is crucial for the early stages of HPV infections and delivery through the host cell.

**Figure 2:**
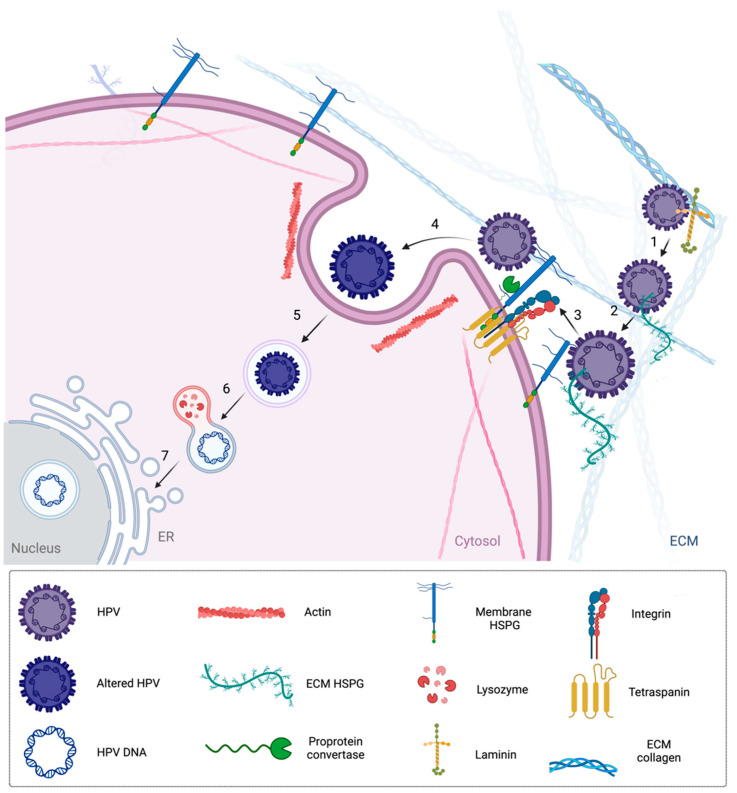
HPV entry through host cells. HPV (type 16) attaches to laminin, free heparan sulfates in the ECM, or membrane (1,2), and the virus changed conformationally via exposing additional amino acids of the L2 N terminus. Then, the virion binds to integrin, which promotes intracellular signaling and further conformational changes (3). Following this, endocytosis of HPV16 takes place through the cytosol (4,5), lysozyme (6), and ER (7) to reach the nucleus [79,80,81] (Created with BioRender.com).

**Figure 3:**
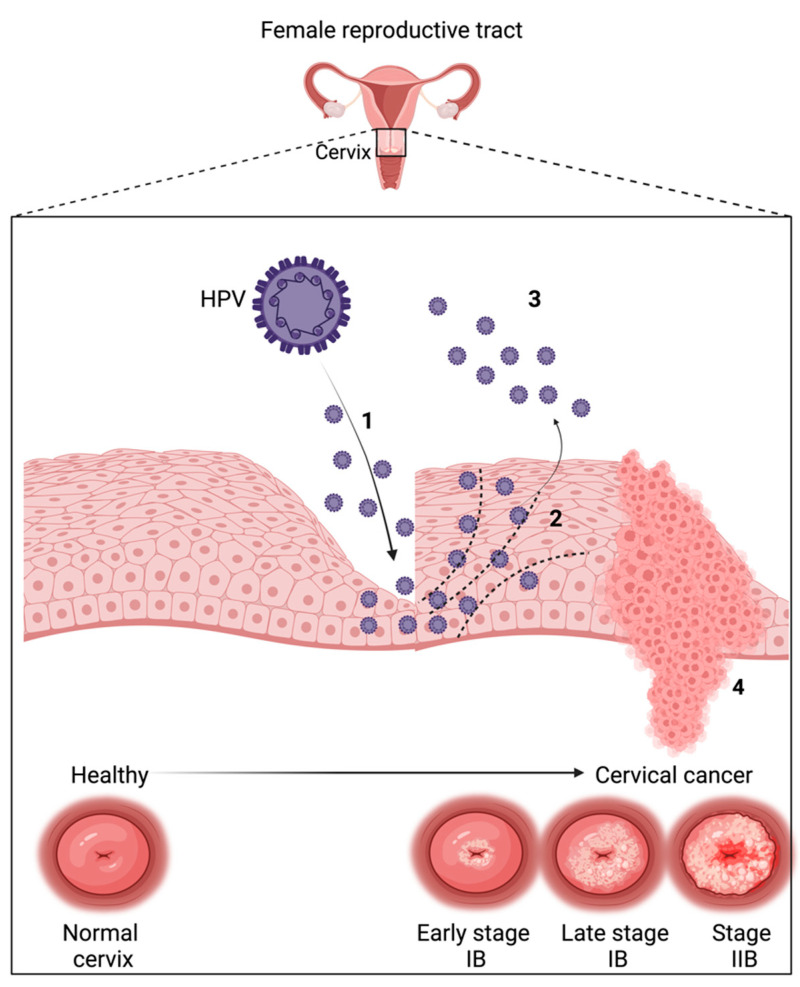
HPV infection and development of cervical cancer. HPV enters the basal cells via micro-abrasions in the cervical layer (1). Through further infection, the viral genome is replicated and progeny virions are formed to initiate a new infection which has a high risk to progress lesions (2,3). Untreated lesions are associated with early gene disruption (E2) and upregulation (E6–E7), which stimulates oncogene expression and invasive cancer development (4) [77]. (Created with BioRender.com).

In HPV infection and cervical cancer development, several sequential steps take place to first infect host cells, and finally, generate carcinogenesis. The life cycle of HPV is initiated by the binding of viral particles to the basal layer of the epithelium (Figure 2). HS chains found on cell membrane proteins are considered receptors for viral entry. HPV uses free HS in the extracellular matrix and in the membrane to bind them and is able to enter through the host cell [79,80,81]. The virus remains stable as an episome with a low copy number in the basal layer. When HPV reaches the host, it uses the host cell replication system for its replication, which is initiated by viral DNA replication [82,83]. Then, the basal layer is divided, and one daughter cell goes away from the basal membrane to be differentiated. The viral DNA is packed and released outside of the epithelium to restart infection. During the HPV infection, E1 and E2 proteins are generally considered the recognition and regulation molecules of early viral progress [84,85]. During the development of cervical cancer related to HPV infection, the HPV genome is integrated through the host genome and HPV has a full life cycle that persists in the host cell. E1 and E2 proteins, which maintain the genes and regulate E6–E7, are generally disrupted during viral integration through the host cell. Furthermore, some tumor-suppressing genes, including p53 and Rb, are inhibited due to the misregulation of E6 and E7, which are associated with different proteins playing a role in the cell cycle, DNA repair, apoptosis, and translation. Uncontrolled cellular proliferation allows cells to bypass cell cycle checkpoints, leading to the development of cervical cancer (Figure 3) [86].

It was shown that bovine Lf inhibited HPV-16 virus-like particle attachment and entry to HaCaT cells [59]. The flow cytometry-based study indicated bovine Lf inhibition on HPV internalization was recorded as dose-dependent and the most effective with IC50 = 35 μg/mL causing around a 50% decrease. Interestingly, being dose-dependent, it was effective in hindering HPV attachment to HaCaT cells. In a similar manner to the previous data, bovine Lf inhibited the binding at lower concentrations than human Lf and it showed a 90% inhibition; HPV-16 uptake was prevented with Lf addition to the host cells and the virus, while pre-incubation cells with HPV during the long periods before Lf addition caused a dramatic decline in the antiviral effect of Lf. Thus, the antiviral effect of Lf was significantly lower when the viral particle was bound to the receptor [59]. Another in vitro study investigated the antiviral effect of goat Lf on HeLa cells, which are derived from cervical cancer, and quantitatively measured the DNA of HPV extracted from these cell pellets using real-time PCR. It was reported that Lf isolated from Etawa goats was highly effective in inhibiting HPV infection in HeLa cells. Notably, the group found that goat Lf exhibited its antiviral activity at 100 μg/mL after 72 h of incubation with HPV cells and increased the cycle threshold value from 26 Ct to 36 Ct [87]. In another study related to bovine Lf usage for genital warts caused by HPV, it was reported that transferosomal Lf effectively improved genital warts [69]. They evaluated the applicability and efficacy of a transferosomal vesicle system with bovine Lf on genital warts. The encapsulation efficiency of transferosomes, which are prepared by reverse-phase evaporation and thin-film hydration, was recorded as 91% for Lf, and the efficacy was measured using the MTT assay on HeLa cells. Importantly, they unveiled that transferosomal Lf improved the IC50 value of HPV inhibition, being ten times more effective than free Lf [69]. Moreover, Mistry and his colleagues demonstrated that not only Lf, but also lactoferricin peptide, which is an *N*-terminal fragment of Lf formed after its digestion by pepsin, was observed with antiviral effects against HPV-5 and HPV-16. Bovine lactoferricin (17–42) had antiviral activity against only the HPV-5 pseudovirus infection on the HaCaT and C33A cell lines. Noticeably, bovine lactoferricin (17–31) was considered the most effective peptide since it showed a higher antiviral effect to HPV-5 and HPV-16 in cell lines among other derivatives [88].

On the other hand, several studies have supported that Lf exerts an anticancer effect against HPV-related cervical cancer [20,89,90]. For instance, in a study, the anticancer effect of bovine Apo-Lf on cervical cancer HeLa cells was examined by analyzing the ROS, NAD+, and GSH concentrations [89]. It was noted that Apo-Lf significantly triggered apoptosis in HeLa cells and regulated pro-apoptotic protein expression, however, diferric bovine Lf was not effective in the concentration from 1 to 12.5 μM for 72 h. Apo-Lf modulated Bax, Bcl-2, Mcl-1, PARP-1, and Sirt-1 molecules at 1.25 μM up to 72 h and triggered apoptosis by the cleavage of ADP-ribose polymerase, reducing NAD+ and activating caspase [89]. In addition, goat Lf has been considered an effective anticancer protein against HeLa cells with cervical cancer. In the related study, the cytotoxicity of goat Lf on the AMN-3 and REF cell lines was evaluated with different concentrations from about 19 to 5000 μg/mL and durations of 1 day, 2 days, and 3 days [70]. Therefore, goat Lf showed an increasing inhibition with the concentration and incubation time. They recorded the highest cytotoxic effect at a concentration of 5000 μg/mL after 3 days at 56.14% [70]. Hence, the studies to date have supported that Lf from different dairy milk sources presents a high therapeutic effect against both HPV infection and its related cervical cancer.

While the range of antiviral agents available for HPV treatment is still limited and varied, some promising options have begun to emerge. For example, cidofovir has been assessed in various studies as a treatment for HPV infections, including persistent cases where conventional therapies have failed. Additionally, other antiviral agents, such as Lf, are being explored. This antiviral agent, available as a 1% topical cream and a 2.5 mg/mL intralesional solution, has shown effectiveness in managing relapsing HPV lesions, including those resistant to other treatments. For topical use, cidofovir was applied daily for 5 days, followed by a 10-day break, with several cycles often leading to a complete resolution of lesions. Intralesional cidofovir has been used successfully for resistant warts, demonstrating its selective antiviral activity against HPV-infected cells with minimal side effects and no reported resistance [91]. Additionally, cidofovir is widely used for managing recurrent respiratory papillomatosis (RRP), where it interferes with viral DNA replication, increasing relapse-free times and reducing the need for surgeries. A study involving 82 adults and 36 children with RRP used cidofovir at concentrations ranging from 2.5 to 7.5 mg/mL, with the higher doses generally yielding better outcomes. Importantly, cidofovir did not induce dysplastic changes in HPV-infected laryngeal tissue in 2% of the patients. Despite its benefits, the use of cidofovir requires careful monitoring due to its potential side effects, and its effectiveness can vary depending on the dose and application method [92]. Therefore, cidofovir is a proven antiviral treatment for HPV with well-established clinical use, demonstrating effectiveness in managing persistent HPV infections and RRP. In contrast, Lf shows promising antiviral and anticancer properties in pre-clinical studies. While Lf exhibits significant antiviral activity and potential anticancer effects, its clinical application is still emerging. Both treatments are effective for managing HPV-related conditions, with cidofovir being more established in clinical practice and Lf representing a potentially valuable alternative for future medicinal and clinical use.

On the other hand, interferon-γ (IFN-γ) is a key Th1 cytokine that enhances cell-mediated immunity and plays a crucial role in the clearance of high-risk HPV, which is associated with cervical cancer [93,94]. A study involving 57 patients with high-risk HPV and mild dysplasia found that IFN-γ positivity was significantly linked to the clearance of HPV after 12 months of follow up, with 93.3% of the IFN-γ-positive patients clearing the infection compared to the 66.7% of IFN-γ-negative patients. Other cytokines (IL-10, IL-6, and TNF-α) did not show significant associations with HPV clearance. The study concluded that IFN-γ might serve as a prognostic marker for HPV clearance, suggesting that its presence is a favorable indicator for the natural regression of HPV-related lesions. However, limitations include the small sample size and reliance on cervical tissue samples, which may not fully represent systemic cytokine responses [94]. A phase 2 study evaluated a vaccine containing synthetic peptides from HPV-16 oncoproteins E6 and E7 in patients with HPV-16-positive, grade 3 vulvar intraepithelial neoplasia (VIN). The vaccine induced significant immune responses, including interferon-γ production, and led to promising clinical outcomes: 25% of the patients achieved complete regression and 35% had partial regression within 3 months. By 12 months, 79% of the patients showed clinical responses. Adverse effects were primarily local reactions at the injection site and flu-like symptoms, with no severe events reported. The vaccine effectively stimulated HPV-16-specific T-cell responses, with interferon-γ production correlating with clinical improvement. Therefore, the increased interferon-γ production due to this vaccination could be the primary reason for the regression in HPV-positive patients [95]. To sum up, cidofovir, IFN-γ, and Lf each offer distinct advantages in managing HPV infections and related conditions. Cidofovir, available as a topical cream or intralesional solution, is effective for persistent and resistant HPV lesions, including recurrent respiratory papillomatosis, with minimal side effects but requires careful monitoring. IFN-γ, a cytokine crucial for enhancing cell-mediated immunity, significantly correlates with HPV clearance and serves as a prognostic marker for lesion regression, though its effectiveness can be limited by sample size and reliance on tissue samples. Lf from bovine and goat sources provides both antiviral and anticancer effects against HPV, with improved efficacy through transferosomal delivery and the potential for inducing apoptosis in HPV-related cervical cancer cells. Each approach offers unique benefits, and their combination could enhance treatment outcomes for HPV-related diseases. Consequently, Lf’s antiviral properties and immune modulation could enhance the effects of cidofovir and IFN-γ, potentially leading to more effective viral clearance, reduced resistance, and improved tissue healing. By targeting different stages of the viral lifecycle, this combination may allow for lower doses of conventional agents, reducing side effects while providing comprehensive protection against HPV and its potential progression to cancer. Lf indeed shows promising antiviral and anticancer properties against HPV, but there are limitations to its use. One major limitation is the variability in efficacy depending on the source of Lf (bovine, goat, etc.), as well as the method of delivery. Additionally, more extensive clinical trials are needed to fully understand the long-term safety and effectiveness of Lf as a therapeutic agent in humans, as most studies to date have been conducted in vitro or in animal models. Despite these factors, Lf is emerging as a potentially competitive clinical therapy agent for HPV due to its dual antiviral and anticancer effects.

### 3.2. Lactoferrin against Other Viral Infections

Many studies have indicated that Lf is a powerful antiviral molecule against various viruses (Table 1) [21,59]. As it can bind to HS chains on HSPGs, which are receptors for HPV entry to the host, it can prevent viral binding and entry [6,8]. Additionally, it can also bind directly to viral glycans specifically and hinder viral entry to the host. Lf also stimulates many different immunity pathways due to its immune-modulatory and anti-inflammatory properties, which enables it to effectively fight against viral infections [18]. Considering Lf’s anticancer properties, it is also possible that it can be an efficient therapeutic to fight cervical cancer. Consequently, the use of Lf could have a high efficiency to inhibit viral infection and might hinder cervical cancer development.

The study performed by Graikini and their colleagues aimed to examine the ability of native Lf isolated from bovine milk and other alternative sources to reduce rotavirus infectivity using a human epithelium model influenced by Caco-2/TC7 cells [100]. Before the neutralization tests, the safety and cytotoxicity concerns regarding bovine Lf were assessed up to 10 mg/mL for cell viability by the same project group previously [105]. After the neutralization tests, which involved pre-treating rotavirus with human, bovine, and camel Lf, rotavirus infection was effectively reduced in a dose-dependent way. At the highest concentration tested (10 mg/mL), the iron-saturated bovine Lf achieved the greatest neutralization activity at 78.7%, with an IC50 of 6.18 mg/mL. However, it was observed that iron saturation did not appear to affect the neutralization activity of the native bovine Lf, which had an IC50 of 6.95 mg/mL. Therefore, treating cells with Lf before RV infection resulted in minimal antirotaviral activity, observed only with native and iron-saturated bovine Lfs. Meanwhile, the highest level of viral infection inhibition achieved was 27.9% with native bovine Lf at a concentration of 10 mg/mL, and 26.9% with iron-saturated bovine LF at a concentration of 6 mg/mL. Finally, applying LF after the virus had adsorbed in the cells demonstrated a moderate effect against RV, with only native bovine Lf and iron-saturated bovine Lf showing a proper inhibition with values of 50.8% and 38.8%, respectively, when administered at a concentration of 10 mg/mL. This study utterly demonstrated that Lf neutralizes rotavirus infection in the Caco-2/TC7 cell model where the cells were differentiated into human enterocytes. Therefore, the observed inhibition extended beyond merely neutralizing viral particles, and also included the partial suppression of intracellular replication in subsequent stages.

In another study, Wróbel and her colleagues aimed at determining the antiviral activity of bovine Lf, specifically against *E. enterovirus* under in vitro conditions; it was noted that there are currently no ongoing studies exploring the antiviral effects of this biocompatible protein against bovine enterovirus [106]. The focus of the study was on evaluating the antiviral properties of bovine Lf against *E. enterovirus*. It was found that only the highest concentration of Lf achieved inhibition of the cytopathic effect, leading to a 63% reduction in virus titre. No direct virucidal effect was detected. Antiviral activity was primarily observed at lower virus doses with significant reductions in viral yield noted during the virus adsorption and post-adsorption stages, especially at the lowest infection dose. No protective effect on cells was demonstrated when Lf was applied prior to infection. Furthermore, the impact of Lf on viral RNA load was less pronounced compared to its effect on extracellular virus titres, with notable reductions being observed. In summary, the study demonstrated that bovine LF effectively reduces viral replication of bovine enterovirus E at both the adsorption and post-adsorption stages, though it does not protect cells directly. The observed reduction in viral RNA and differences in mechanism compared to human enteroviruses highlight the need for further research. Despite lacking cellular protection, bovine Lf’s biocompatibility and antiviral properties suggest potential benefits, especially for young animals receiving it through milk.

In a separate study by a team led by Andreu, liposomal bovine Lf’s potential antiviral activity was demonstrated in vitro against human coronavirus HCoV-229E and SARS-CoV-2 pseudoviruses, compared to non-liposomal bovine Lf [36]. The antiviral effects of liposomal lactoferrin (LL) were assessed against SARS-CoV-2 pseudoviruses and HCoV-229E, alongside free lactoferrin (FL). MTT assays were used in this study to identify non-cytotoxic concentrations for LL and FL in cell lines like Huh-7 and ACE2 + A549, showing LL’s higher cytotoxicity and requirement for lower doses. In vitro, the infection rates of both viruses were significantly reduced by LL at doses where FL was ineffective. Viral infection was decreased by over 50% by LL at concentrations as low as 10^−3^% (*w*/*v*), highlighting its superior antiviral potency. In human lung tissue (HLT) cells, antiviral activity was demonstrated by LL, but with increased cytotoxicity, limiting its therapeutic window. These findings underscore the potential of LL as a potent antiviral agent HCoV-229E and SARS-CoV-2 by blocking viral entry and modulating immune responses, with its enhanced efficacy attributed to encapsulation. However, the clinical application of LL may be limited by its cytotoxicity in sensitive tissues, necessitating possible further optimization and delivery testing [33].

As mentioned, various viral infections and antiviral mechanisms of Lf have been investigated. In recent studies, the potent effects of Lf as conjugated nanoparticles have been proposed and studied as a new concept of a new antiviral agent. In this regard, the most studied and used nanoparticles are gold nanoparticles (AuNPs) and silver nanoparticles (AgNPs) [107,108]. AgNPs have shown interactions with the disulfide bonds of glycoproteins and proteins of potent harmful microorganisms, namely fungi, bacteria, and viruses [108]. As for frequently used AuNPs, a modification performed with mercaptoethane sulfonate (Ag-MES) resulted in the inhibition of viral infections caused by HSV-1 [107]. The inert and non-toxic nature of AuNPs prevent them from influencing further cellular functions, whereas AgNPs are capable of releasing Ag ions and play roles in cellular functions [109,110]. AgNPs demonstrate low antiviral effects in the metabolism solely, but AgNPs with mercaptoethane sulfonate (Ag-MES) were modified by Baram-Pinto and resulted in a mimicry of the HS receptors of host cells blocking HSV-1 infection [111]. As a concept of novel antiviral agents, the exploitation of nanoparticles allowed for targeting biological sites in both active and passive regards [96]. The nanoparticles and their utilization in viral infections will possess a crucial potential in the future, as the NPs are being developed continuously and the modifications are variable.

The interaction between NPs and proteins enables them to perform specific biological activities [96]. In this regard, by using AgNPs and bLf, Nayak and her colleagues concluded that bLf could be absorbed by the AgNP surface by weak chemical interactions such as van der Waals and hydrogen bonds, allowing proteins to maintain their conformation and stability. Additionally, bLf aided in the diminishing of the cytotoxic effects of AgNPs [112]. In another study, hLf-supplemented gold and silver nanoparticles allowed the HSV-2 infection by directly inhibiting viral particles [97]. Lf-conjugated to nanometals is proposed to bind to HSPGs much more efficiently and provide a barrier for HSV-2 infection. Based on this proposition, in vivo experiments were designed and concluded that all Lf conjugates were significantly efficient than sole Lf in the early stages of infection and showed extra immunomodulatory functions. All Lf-conjugated nanomaterials promoted the levels of cytokines and chemokines radically in vaginal cell tissues [97]. Along with this study, hLf-conjugated AuNPs and AgNPs were tested on HCoV-229-e in vitro by using MRC-5 cells. The study had concluded that the inhibition of HCoV-2209e virus depended on the type of metal and the size of NPs, and Lf-conjugated 30 nm silver nanoparticles showed the best results. Altogether, the experiments have proven that Lf-conjugated NPs of metals such as silver and gold should be considered as a reliable source of a new class of antivirals. Knowing the mechanism and limitations of Lf, novel methodologies and approaches can be designed in the example of nanomaterials. Sufficient and appropriate modifications to Lf may lead to more efficient and direct interactions with viral microorganisms and their compartments [96,113]. It is also remembered that pharmacological formulations are required to improve to efficiently and safely use Lf-conjugated NPs and keep them at stable conditions for Lf conjugation.

Iron stacking in the liver in a chronic manner promotes hemochromatosis related to crucial tissue damage, cirrhosis, and hepatocarcinoma (HCC). As a result, ROS are produced by the excess amount of iron and may lead to inflammation hindering physiological and essential hepatic functions [113]. Viral liver infections (maintained by the presence of iron) may provoke severe levels of liver damage. Since the iron-binding nature of Lf is well-known, it is assumed that Lf can perform the inhibition of viral infections as an efficient treatment. Anti-inflammatory functions of Lf may result in the modulation of proteins playing roles in iron homeostasis, such as ferroportin (Fpn) and hepcidin [114,115]. Indeed, Lf can directly increase the relative levels of Fpn and lead to the decrease in iron overload, degrading viral particles and host cells from all essential compartments [115]. Iron proteins are regulated by the presence of expression levels and are dependent on the virus type and the stage of infection [116]. As for hepatitis B virus (HBV), the current studies indicate a common association in iron proteins in long-term HBV patients, a global scale of the positive alteration in serum iron levels and Ftn. HBV groups in current studies perform approximate serum heptidin levels in their absolute values [117,118]. In summary, HBV and other viruses cause viral liver infections and Lf can influence the severity of hepatic diseases as a potential anti-inflammatory and iron-modulator molecule.

## 4. Conclusions

Lf, as a versatile whey protein, is largely abundant in different dairy milk sources including bovine and milk with their derived products, enabling such dairy foods to benefit human health and protect it against different viral infections. Lf, with its crucial biological functions, is considered a promising therapeutic protein to combat HPV infection and other viral infections which cause serious health problems worldwide. Particularly, the antiviral, anti-inflammatory, and immune-modulatory effects of Lf make it a potential inhibitory molecule considered in many viral infection treatment studies by hindering the viral entry process. As Lf can inhibit the binding of the virus to host receptors for its entry and viral infection in the beginning of the viral life cycle, it is an effective antiviral protein even at early infection. Thus, Lf in dairy foods is promising an efficient therapy against HPV and other viral infections because of its functions on viral attachment and infection. Yet further experimental and clinical studies are needed to better understand Lf’s exact molecular mechanism and effects on viral infections.

## Figures and Tables

**Table 1:** Examples of different effects of Lf from various dairy species on HPV and other viral infections.

Effect	Dairy Species	Study	Reference
Binding to HPV-16 virus-like particles and inhibits their internalization to HaCaT cells	Bovine	In vitro	[59]
Bovine lactoferrin derivative lactoferricin inhibits the infection of HPV-16 and HPV-5 in HaCaT and C33A cells	Bovine	In vitro	[88]
Improvement of genital warts caused by HPV via the system of transdermal delivery	Bovine	In vitro	[69]
Viral replication and infection inhibition in HeLa cells	Goat	In vitro	[87]
Stimulation of apoptosis in HeLa cells by triggering oxygen radicals and glutathione level	Bovine	In vitro	[89]
Anticancer activity and cytotoxicity effect on AMN3 and REF cells	Goat	In vitro	[70]
Triggering to increase the level of antiviral cytokines and chemokines in the vaginal tissue	Bovine	In vitro	[96]
The prevention of HSV-2 infection in AgNP and AuNP	Bovine	In vitro, in vivo	[97]
The prevention of SARS-CoV-2 infection by combining RBD and inhibiting coronaviruses’ RdRp activity	Bovine	In vitro	[98]
Increase in the antiviral response associated with the double-stranded RNA-stimulated signaling pathway	Bovine	In vitro	[99]
The neutralization of rotavirus infection in Caco-2/TV7 cells differentiated as human enterocytes	Bovine, camel	In vitro	[100]
Inhibition of hepatitis B virus DNA in HepG2 cells	Bovine	In vitro	[101]
Lessened symptoms in individuals with mild or moderate COVID-19	Hen egg white bovine colostrum mixture	In vivo	[102]
CoV-2-Wtpv entry suppression into ACE2-expressing cells	Bovine	In vitro	[103]
Inhibition of activity and secretion of 110-Mh metalloprotease from *M. haemolytica* A2	Bovine	In vitro	[104]
